# Supplementary material for: Sex disparities in adult obesity prevalence across 47 African countries: A cross-sectional descriptive study
Source: PLoS One. 2025 Dec 22;20(12):e0339595. doi: 10.1371/journal.pone.0339595 (PMC12721531; doi:10.1371/journal.pone.0339595)
Supplement: Attachment — Submitted filename: OsagbemiPONE-D-25-39220.docx [file pone.0339595.s001.docx]

PLOS ONE

Sex disparities in adult obesity prevalence (BMI ≥30) across 47 African countries: a cross-sectional descriptive study using the World Health Organization health equity

assessment toolkit

--Manuscript Draft—

Specific comments:

1. The study TITLE need not reflect the WHO tool utilized for the study. It suffices to be in the methodology

2. Tables are to be properly labelled and should not spill into pages

3. Technical epidemiological terms such as significant (may connote statistical test), gaps (may connote unmet needs) have been inappropriately used in the text. Alternatives have been included in the review.

4. Elements of discussion should not come up in the results section but taken to the appropriate sections of discussion and conclusion. These have been indicated (green highlights) in situ of this review.

5. A separate subsection to make concrete actionable stakeholder - targeted recommendations is needed

| Manuscript Number: | PONE-D-25-39220 |
| --- | --- |
| Article Type: | Research Article |
| Full Title: | Sex disparities in adult obesity prevalence (BMI ≥30) across 47 African countries: a cross-sectional descriptive study using the World Health Organization health equity assessment toolkit |
| Short Title: | adult obesity prevalence (BMI ≥30) across 47 African countries |
| Corresponding Author: | Augustus Osborne  Institute for Development  Freetown, SIERRA LEONE |
| Keywords: | Obesity; sex disparities; Africa; non-communicable diseases; public health |
| Abstract: | Background  Obesity is a growing public health concern in Africa, with evidence suggesting significant disparities between men and women. Understanding the prevalence and sex differences in obesity across African countries is critical for informing equityfocused interventions. This study examined the prevalence of adult obesity (body mass index [BMI] ≥30) across 47 African countries using sex-disaggregated data, and to assess the magnitude of sex disparities in obesity prevalence as a basis for equityfocused public health interventions.  Methods  I conducted a cross-sectional descriptive study using nationally representative, sexdisaggregated data from 47 African countries, primarily for the year 2022, obtained from the World Health Organization Health Equity Assessment Toolkit (HEAT). Adults aged 18 years and older with available, recent data were included. The primary outcome was the country-level prevalence of adult obesity (body mass index [BMI] ≥30) disaggregated by sex. The secondary outcome was the absolute difference in obesity prevalence between women and men within each country.  Results  Obesity prevalence among adults varied widely across African countries. South Africa had the highest prevalence at 30.8%, followed by Eswatini (30.1%) and Seychelles (29.4%), while Ethiopia (2.8%), Madagascar (4.3%), and Eritrea (4.8%) reported the lowest rates. Across nearly all countries, women exhibited significantly higher obesity prevalence than men. In South Africa, female obesity prevalence was 45.8%, compared to 13.9% in men a difference of 31.8 percentage points. Large sex  disparities were also observed in Eswatini (difference: 28.8), Mauritania (26.1), Lesotho  (24.2), Equatorial Guinea (21.8), and Seychelles (19.4). In contrast, only a few countries, such as Burundi, Chad, and Madagascar, showed negligible or slightly higher obesity prevalence among men.  Conclusion  The findings reveal that adult obesity is a significant and growing public health concern in Africa, with marked heterogeneity in prevalence between countries and an overwhelming burden among women. These disparities reflect the sociocultural, economic, and biological factors influencing obesity risk, including urbanization, dietary transitions, and gender norms. The pronounced gender gap underscores the need for context-specific, gender-sensitive interventions and policies to effectively address obesity and its health consequences. Policymakers and health practitioners should prioritize multisectoral strategies that promote healthy diets, physical activity, and |

|  | address the unique barriers faced by women. Enhanced surveillance and research are also needed to further elucidate the determinants of obesity and to monitor progress toward reducing health inequities across the continent. |
| --- | --- |
| Order of Authors: | Augustus Osborne |
| Additional Information: |  |
| Question | Response |
| Financial Disclosure  Enter a financial disclosure statement that describes the sources of funding for the work included in this submission. Review the [submission guidelines](http://journals.plos.org/plosone/s/submission-guidelines#loc-financial-disclosure-statement) for detailed requirements. View published research articles from [PLOS ONE](https://journals.plos.org/plosone/) for specific examples.  This statement is required for submission and will appear in the published article if the submission is accepted. Please make sure it is accurate. | The author(s) received no specific funding for this work. |
| Funded studies Enter a statement with the following details: - Initials of the authors who received each award - Grant numbers awarded to each author - The full name of each funder - URL of each funder website - Did the sponsors or funders play any role in the study design, data collection and analysis, decision to publish, or preparation of the manuscript? |  |
| Did you receive funding for this work? |  |
| Competing Interests  Use the instructions below to enter a competing interest statement for this submission. On behalf of all authors, disclose any [competing interests](http://journals.plos.org/plosone/s/competing-interests) that could be perceived to bias this work—acknowledging all financial support and any other relevant financial or nonfinancial competing interests.  This statement is required for submission | The authors have declared that no competing interests exist. |

| and will appear in the published article if the submission is accepted. Please make sure it is accurate and that any funding sources listed in your Funding Information later in the submission form are also declared in your Financial Disclosure statement.  View published research articles from [PLOS ONE](http://journals.plos.org/plosone/) for specific examples. |  |
| --- | --- |
| NO authors have competing interests Enter: The authors have declared that no competing interests exist. Authors with competing interests Enter competing interest details beginning with this statement: I have read the journal's policy and the authors of this manuscript have the following competing interests: [insert competing interests here] |  |
| * typeset |  |
| Ethics Statement  Enter an ethics statement for this submission. This statement is required if the study involved:   - Human participants - Human specimens or tissue - Vertebrate animals or cephalopods - Vertebrate embryos or tissues - Field research   Write "N/A" if the submission does not require an ethics statement.  General guidance is provided below. Consult the [submission guidelines](http://journals.plos.org/plosone/s/submission-guidelines#loc-guidelines-for-specific-study-types) for detailed instructions. Make sure that all information entered here is included in the Methods section of the manuscript. | This study used publicly available, anonymized data from the World Health  Organization Global Health Observatory and the Health Equity Assessment Toolkit. As no primary data were collected and no individual-level identifiers were used, ethical approval and informed consent were not required. |

Format for specific study types

Human Subject Research (involving human

participants and/or tissue)

Give the name of the institutional review

board or ethics committee that approved the

study

•

Include the approval number and/or a

statement indicating approval of this

research

•

Indicate the form of consent obtained

(

written/oral) or the reason that consent was

not obtained (e.g. the data were analyzed

anonymously)

•

Animal Research (involving vertebrate

animals, embryos or tissues)

Provide the name of the Institutional Animal

Care and Use Committee (IACUC) or other

relevant ethics board that reviewed the

study protocol, and indicate whether they

approved this research or granted a formal

waiver of ethical approval

•

Include an approval number if one was

obtained

•

If the study involved

non-human primates

,

add

additional details

about animal welfare

and steps taken to ameliorate suffering

•

If anesthesia, euthanasia, or any kind of

animal sacrifice is part of the study, include

briefly which substances and/or methods

were applied

•

Field Research

Include the following details if this study

involves the collection of plant, animal, or

other materials from a natural setting:

Field permit number

•

Name of the institution or relevant body that

granted permission

•

Data Availability

Authors are required to make all data

underlying the findings described fully

available, without restriction, and from the

time of publication. PLOS allows rare

exceptions to address legal and ethical

concerns. See the

[PLOS Data Polic](http://journals.plos.org/plosone/s/data-availability)

[y](http://journals.plos.org/plosone/s/data-availability)

and

[FA](http://journals.plos.org/plosone/s/data-availability#loc-faqs-for-data-policy)

[Q](http://journals.plos.org/plosone/s/data-availability#loc-faqs-for-data-policy)

for detailed information.

Yes - all data are fully available without restriction

| A Data Availability Statement describing where the data can be found is required at submission. Your answers to this question constitute the Data Availability Statement and will be published in the article, if accepted. |  |
| --- | --- |
| Important: Stating ‘data available on request from the author’ is not sufficient. If your data are only available upon request, select ‘No’ for the first question and explain your exceptional situation in the text box. |  |
| Do the authors confirm that all data underlying the findings described in their manuscript are fully available without restriction? |  |
| Describe where the data may be found in full sentences. If you are copying our sample text, replace any instances of XXX with the appropriate details.   - If the data are held or will be held in a public repository, include URLs, accession numbers or DOIs. If this information will only be available after acceptance, indicate this by ticking the box below. For example: All XXX files   are available from the XXX database (accession number(s) XXX, XXX.).   - If the data are all contained within the manuscript and/or Supporting Information files, enter the following: All relevant data are within the manuscript and its Supporting Information files. - If neither of these applies but you are able to provide details of access elsewhere, with or without limitations, please do so. For example:   Data cannot be shared publicly because of [XXX]. Data are available from the XXX Institutional Data Access / Ethics Committee (contact via XXX) for researchers who meet the criteria for access to confidential data.  The data underlying the results presented in the study are available from (include the name of the third party | The data used in this study was obtained from the World Health Organization Global  Health Observatory (WHO GHO) and the WHO Health Equity Assessment Toolkit (HEAT). These data are publicly available and can be accessed without restriction from the WHO Global Health Observatory website (https://www.who.int/data/gho) and the WHO HEAT platform (https://www.who.int/data/inequality-monitor/assessment_toolkit). The author confirm that all interested researchers can access these data in the same manner as the authors, and no special access privileges were required. |

| and contact information or URL).  • This text is appropriate if the data are owned by a third party and authors do not have permission to share the data.  * typeset |  |
| --- | --- |
| Additional data availability information: |  |

[Click here to access/download;Manuscript;Manuscript_Obesity_adults_ in](https://www2.cloud.editorialmanager.com/pone/download.aspx?id=41284822&guid=70a9697e-0d21-419a-a688-ba02d538bf8b&scheme=1)

Sex disparities in adult obesity prevalence (BMI ≥30) across 47 African countries: a crosssectional descriptive study using the World Health Organization health equity assessment toolkit

Augustus Osborne ^1*^

1. Institute for Development, Western Area, Freetown, Sierra Leone

*Correspondence: augustusosborne2@gmail.com

AO: augustusosborne2@gmail.com

# Abstract

**Background:**

Obesity is a growing public health concern in Africa, with evidence suggesting significant disparities between men and women. Understanding the prevalence and sex differences in obesity across African countries is critical for informing equity-focused interventions. This study examined the prevalence of adult obesity (body mass index [BMI] ≥30) across 47 African countries using sex-disaggregated data, and to assess the magnitude of sex disparities in obesity prevalence as a basis for equity-focused public health interventions.

**Methods:**

I conducted a cross-sectional descriptive study using nationally representative, sexdisaggregated data from 47 African countries, primarily for the year 2022, obtained from the World Health Organization Health Equity Assessment Toolkit (HEAT). Adults aged 18 years and older with available, recent data were included. The primary outcome was the country-level prevalence of adult obesity (body mass index [BMI] ≥30) disaggregated by sex. The secondary outcome was the absolute difference in obesity prevalence between women and men within each country.

# Results

Obesity prevalence among adults varied widely across African countries. South Africa had the highest prevalence at 30.8%, followed by Eswatini (30.1%) and Seychelles (29.4%), while Ethiopia (2.8%), Madagascar (4.3%), and Eritrea (4.8%) reported the lowest rates. Across nearly all countries, women exhibited significantly higher obesity prevalence than men. In South Africa, female obesity prevalence was 45.8%, compared to 13.9% in men a difference of 31.8 percentage points. Large sex disparities were also observed in Eswatini (difference: 28.8), Mauritania (26.1), Lesotho (24.2), Equatorial Guinea (21.8), and Seychelles (19.4). In contrast, only a few countries, such as Burundi, Chad, and Madagascar, showed negligible or slightly higher obesity prevalence among men.

# Conclusion

The findings reveal that adult obesity is a significant and growing public health concern in Africa, with marked heterogeneity in prevalence between countries and an overwhelming burden among women. These disparities reflect the sociocultural, economic, and biological factors influencing obesity risk, including urbanization, dietary transitions, and gender norms. The pronounced gender gap underscores the need for context-specific, gender-sensitive interventions and policies to effectively address obesity and its health consequences. Policymakers and health practitioners should prioritize multisectoral strategies that promote healthy diets, physical activity, and address the unique barriers faced by women. Enhanced surveillance and research are also needed to further elucidate the determinants of obesity and to monitor progress toward reducing health inequities across the continent.

**Keywords:** Obesity, sex disparities, Africa, non-communicable diseases, public health

**Introduction**

Obesity has emerged as one of the most pressing public health challenges of the 21st century, contributing substantially to global morbidity and mortality [1,2]. The adverse health consequences of obesity are well-established, encompassing an increased risk for cardiovascular diseases, type 2 diabetes, certain cancers, musculoskeletal disorders, and a range of other non-communicable diseases (NCDs) [3]. Despite growing awareness and the implementation of various public health initiatives, the prevalence of obesity continues to rise worldwide, with low- and middle-income countries (LMICs) experiencing a particularly rapid escalation of the epidemic [4]. In Africa, the burden of obesity is increasing at an alarming rate, largely driven by urbanization, economic transitions, and shifts in dietary and physical activity patterns [5]. Understanding the patterns and determinants of obesity especially through sex-disaggregated data is essential for addressing health inequities and designing targeted interventions to curb the rising tide of obesity and its associated health burdens.

Globally, the World Health Organization (WHO) estimates that more than 650 million adults are classified as obese (BMI ≥30), with the prevalence of obesity nearly tripling since 1975 [6]. While obesity was once considered a problem exclusive to high-income countries, it is now recognized as a growing concern in LMICs, including those in Africa [7]. Notably, obesity prevalence is not uniformly distributed across populations; significant disparities exist between men and women, with women generally exhibiting higher rates of obesity than men in most African countries [8]. According to recent WHO data, the prevalence of obesity among adult women in Africa often exceeds that of men by a substantial margin, reflecting complex interactions between biological, sociocultural, and environmental factors [9]. In several African countries, more than one in five adult women are classified as obese, while the prevalence among men remains considerably lower [10]. These sex disparities underscore the urgent need for region- and sex-specific data to inform effective public health strategies and policy responses.

Previous research has extensively documented the health impacts of obesity and identified key sociodemographic determinants, including age, sex, education, income, and place of residence [11-13]. However, much of the existing literature has focused on high income settings or provided global estimates, with limited attention to the African context. Moreover, there is a notable lack of consistent, up-to-date, and comparable data on obesity prevalence across African countries particularly sex-disaggregated data that can illuminate gender-specific patterns and inequalities [14]. Although some studies have examined obesity trends in individual African countries or subregions, these efforts have often been limited by methodological inconsistencies, data gaps, and a lack of comprehensive geographic coverage [15]. As a result, the true magnitude and distribution of obesity prevalence across Africa especially in relation to sex disparities remain poorly understood.

The African context presents unique challenges and opportunities for addressing the obesity epidemic. Cultural norms, social expectations, and traditional beliefs regarding body image and gender roles play a significant role in shaping obesity patterns among African populations [16]. In many African societies, larger body size particularly among women has historically been associated with health, wealth, and social status, contributing to higher obesity prevalence among women [17]. However, these cultural attitudes are not uniform across the continent and vary widely by region, country, and urban versus rural residence. For example, urbanization and economic development have contributed to rising obesity rates in both men and women in countries such as South Africa and Egypt, while rural populations in countries like Ethiopia and Niger continue to exhibit relatively low prevalence [18]. Additionally, the nutrition transition characterized by increased consumption of energy-dense, processed foods and reduced physical activity has accelerated the rise in obesity, particularly among women who may face unique barriers to physical activity and healthy eating. Weak policy responses limited public health infrastructure, and insufficient surveillance systems have further complicated efforts to address obesity in many African countries. Understanding the sex-specific patterns of obesity is therefore critical for designing effective, culturally appropriate interventions that address the unique challenges faced by African populations.

This study provides a comprehensive, sex-disaggregated analysis of adult obesity prevalence (BMI ≥30) across 47 African countries using data from the World Health

Organization (WHO) Health Equity Assessment Toolkit (HEAT) [19]. By leveraging this standardized and validated data source, the study addresses a critical gap in the literature: the lack of reliable, up-to-date, and comparable estimates of obesity prevalence across the African continent. In addition to assessing overall and sex-specific obesity prevalence, this study aims to identify countries with the greatest need for targeted interventions and to highlight data gaps in existing surveillance systems. The findings offer valuable insights into the magnitude of gender disparities in obesity prevalence, emphasizing the need for gender-sensitive public health policies and interventions. Furthermore, this study contributes to the global effort to monitor and reduce obesity, aligning with the WHO Global Action Plan for the Prevention and Control of NCDs and the Sustainable Development Goals (SDGs), particularly Goal 3, which seeks to ensure healthy lives and promote well-being for all at all ages.

# Methods Study design and period

This study adopted a cross-sectional design to estimate the sex-specific prevalence of adult obesity (BMI ≥30) across 47 African countries. The cross-sectional approach was selected as it allows for the assessment of health outcomes and disparities at a specific point in time, offering a snapshot of obesity patterns and sex differences during the study period. The analysis focused on the most recent available data for each country, primarily from the year 2022, as compiled by the World Health Organization (WHO). Countries were included based on the availability of up-to-date and comparable obesity data in the WHO Health Equity Assessment Toolkit (HEAT). Countries lacking sufficient or recent data were excluded to ensure the robustness and comparability of the results.

# Data source

Data for this study were sourced from the WHO Health Equity Assessment Toolkit (HEAT), an interactive software tool that enables users to examine health inequalities using disaggregated data from WHO Member States. HEAT provides access to a wide range of health indicators, including those related to non-communicable diseases such as obesity, and is recognized for its rigorous data quality and standardization. The platform draws on nationally representative sources, including Demographic and Health Surveys (DHS), WHO Stepwise Approach to Surveillance (STEPS), and other WHO-approved surveys. These surveys employ robust sampling techniques to ensure data accuracy and representativeness, making them reliable for monitoring health indicators like obesity prevalence.

Out of 54 African countries, 47 were included in this analysis. Exclusion of certain countries was due to the absence of sex-disaggregated obesity prevalence data in the HEAT platform. This selection criterion ensured that the analysis relied on high-quality, standardized, and comparable data across countries, reducing the risk of bias from incomplete or inconsistent reporting.

# Outcome measure and dimension of inequality

The principal outcome of interest was the prevalence of obesity, defined as the proportion of adults (aged 18 years and above) with a body mass index (BMI) of 30 or higher at the time of the survey. The data were disaggregated by sex (male and female) to enable a detailed assessment of sex-based differences in obesity prevalence.

Focusing on sex as an equity dimension is essential for understanding how obesity is distributed between men and women and for identifying disparities that may warrant targeted interventions. The main measure of inequality reported was the absolute difference in obesity prevalence between men and women in each country. Relative measures, such as the male-to-female obesity ratio, were not included in this analysis, in line with the study’s primary objective of providing clear, sex-specific prevalence estimates. Future research may consider relative measures to further contextualize these findings.

# Data analysis

All analyses were performed within the WHO HEAT platform, which offers a streamlined interface for exploring and comparing health inequalities. HEAT includes several analytical modules; for this study, the "compare inequality" module was used to generate sex disaggregated estimates of adult obesity prevalence for each of the 47 countries.

The analytical process involved the following steps:

- Accessing the WHO HEAT software via the WHO website and selecting the "compare inequality" module.
- Choosing the indicator "obesity prevalence (BMI ≥30) among adults" from the available health indicators.
- Selecting sex as the dimension of inequality to disaggregate the data.
- Extracting sex-specific prevalence estimates for each country, along with the absolute difference in prevalence between males and females. A negative difference indicates higher obesity prevalence among women compared to men.
- HEAT automatically calculates 95% confidence intervals for each estimate, accounting for sampling variability and ensuring the reliability of results. The platform applies standardized statistical methods, such as sample weighting and stratification, to produce nationally representative estimates.
- Results are provided in both tabular and graphical formats, including bar charts and scatter plots, to facilitate interpretation and highlight the magnitude of sex disparities in obesity prevalence across countries. Data were exported from HEAT and organized into tables for inclusion in the manuscript.

Countries or subgroups without reliable, sex-disaggregated obesity prevalence data were excluded from the analysis. No imputation was performed, as the HEAT platform uses only validated, pre-analyzed data from WHO-endorsed sources. This approach ensures the analysis is based on high-quality and complete data, minimizing the risk of bias from missing information.

The HEAT platform ensures comparability and standardization by harmonizing indicator definitions, survey methodologies, and applying rigorous quality control procedures. This includes adjusting for sampling design and response rates to ensure estimates are representative and comparable across different settings.

No additional statistical analyses or modelling were conducted outside of the HEAT environment. All estimates and calculations are based on the WHO’s standardized procedures, ensuring transparency, reproducibility, and alignment with global best practices for health inequality monitoring. By utilizing the HEAT toolkit, this study benefits from the WHO’s methodological expertise, providing reliable and comparable estimates of sex disparities in adult obesity across Africa.

# Ethics approval and considerations

This study used publicly available, anonymized data from the World Health Organization Global Health Observatory and the Health Equity Assessment Toolkit. As no primary data were collected and no individual-level identifiers were used, ethical approval and informed consent were not required.

# Results Prevalence of obesity among adults in Africa

Table 1 displays the prevalence of obesity among adults across various African countries, highlighting substantial differences in obesity rates and distribution. South Africa has the highest prevalence of adult obesity at 30.8 percent, making it the country with the most widespread obesity problem in the region. Eswatini and Seychelles also report notably high rates, with 30.1 percent and 29.4 percent, respectively, indicating that nearly one in three adults in these countries is classified as obese. Algeria follows with a prevalence of 23.8 percent, while Mauritania and Lesotho both show high rates at 22.7 percent and 21 percent, respectively. Gabon and Botswana also fall into this upper range, with obesity rates of 21 percent and 18.3 percent respectively.

Mauritius, Namibia, Liberia, Equatorial Guinea, Comoros, and Sao Tome and Principe report obesity prevalence between 16 and 19 percent, suggesting that obesity is a considerable public health concern in these countries This should be removed to discussion section as well as part of conclusion. Cabo Verde, Cameroon, Gambia, and Zimbabwe have moderate levels of obesity, ranging from 14.2 to 15.8 percent, indicating that a considerable portion of the adult population in these countries is affected.Discussion

A number of countries, including Ghana, Nigeria, United Republic of Tanzania, Kenya, Benin, Guinea-Bissau, Mali, Togo, and Côte d'Ivoire, shows obesity prevalence rates in the range of 11 to 13 percent. These figures suggest that while obesity is not as widespread as in the highest-prevalence countries, it remains a notable health issue that requires attention. This should be removed to discussion section as well as part of conclusion

Countries such as Angola, Burkina Faso, Chad, Democratic Republic of the Congo, Sierra Leone, South Sudan, Mozambique, Senegal, Malawi, and Zambia report lower to moderate obesity rates, generally between 6 and 11 percent. These countries may face emerging challenges related to obesity as lifestyles and diets continue to change. This should be removed to discussion section as well as part of conclusion

The lowest obesity prevalences are observed in Ethiopia (2.8 percent), Eritrea (4.8 percent), Rwanda (4.9 percent), Madagascar (4.3 percent), and Burundi (5 percent). These low rates may reflect differences in socioeconomic factors, dietary patterns, physical activity, or public health interventions This should be removed to discussion section as well as part of conclusion. Niger (6 percent) and Central African Republic (9.3 percent) also fall on the lower end of the spectrum.

# Table 1: Estimate of obesity prevalence among adults in Africa

| **Country** | **obesity prevalence (%)** |
| --- | --- |
| South Africa | 30.8 |
| Algeria | 23.8 |
| Angola | 11.5 |
| Benin | 11.2 |
| Botswana | 18.3 |
| Burkina Faso | 6.7 |
| Burundi | 5.0 |
| Cabo Verde | 15.8 |
| Cameroon | 14.9 |
| Central African Republic | 9.3 |
| Chad | 6.7 |
| Comoros | 16.3 |
| Congo | 8.5 |
| Cote d'Ivoire | 11.6 |
| Democratic Republic of the  Congo | 6.6 |
| Equatorial Guinea | 17.7 |
| Eritrea | 4.8 |
| Eswatini | 30.1 |

| Ethiopia | 2.8 |
| --- | --- |
| Gabon | 21.0 |
| Gambia | 14.9 |
| Ghana | 12.9 |
| Guinea | 9.5 |
| Guinea-Bissau | 11.5 |
| Kenya | 12.4 |
| Lesotho | 21.0 |
| Liberia | 17.0 |
| Madagascar | 4.3 |
| Malawi | 7.7 |
| Mali | 11.4 |
| Mauritania | 22.7 |
| Mauritius | 19.2 |
| Mozambique | 10.3 |
| Namibia | 17.0 |
| Niger | 6.0 |
| Nigeria | 12.4 |
| Rwanda | 4.9 |
| Sao Tome and Principe | 16.5 |
| Senegal | 10.2 |
| Seychelles | 29.4 |
| Sierra Leone | 7.1 |
| South Sudan | 8.6 |
| Togo | 11.6 |
| Uganda | 7.9 |
| United Republic of Tanzania | 12.6 |
| Zambia | 11.1 |
| Zimbabwe | 14.2 |

296	**TABLES ARE TO BE PROPERLY LABELLED AND SHOULD NOT SPILL INTO PAGES**

# 297 Sex disaggregated obesity prevalence among adults in Africa

Table 2 presents a comprehensive overview of adult obesity prevalence in African countries for 2022, disaggregated by sex and accompanied by confidence intervals. The data reveal substantial disparities between male and female obesity rates, with women consistently exhibiting higher prevalence across nearly all countries.

In South Africa, the contrast between sexes is particularly pronounced. Female obesity prevalence stands at 45.8 percent (confidence interval: 41.5 to 50.3 percent), more than three times higher than the male prevalence of 13.9 percent (confidence interval: 11.1 to 17.1 percent). This significant variation highlights the gendered nature of obesity in the country, where women are disproportionately affected. This should be removed to discussion section as well as part of conclusion

Similar trends are observed in Algeria, where female obesity prevalence is put at 32.3 percent (confidence interval: 27.1 to 37.7 percent), compared to 15.5 percent (confidence interval: 11.6 to 20.0 percent) among men. Eswatini also demonstrates a wide disparity, with 43.9 percent of women (confidence interval: 37.2 to 50.7 percent) classified as obese, versus 15 percent of men (confidence interval: 10.4 to 20.4 percent).

Several countries, including Botswana, Seychelles, Mauritania, and Equatorial Guinea, show female obesity prevalences exceeding 25 percent, while male rates remain below 21 percent. For example, in Seychelles, 39.6 percent of women (confidence interval: 31.8 to 47.4 percent) are obese, compared to 20.2 percent of men (confidence interval: 13.7 to 27.5 percent). In Botswana, female obesity stands at 27.2 percent (confidence interval: 21.4 to 33.6 percent), while male prevalence is significantly lower at 8.4 percent (confidence interval: 5.1 to 12.8 percent). Mauritania and Equatorial Guinea also exhibit similar patterns, with female obesity rates of 35 percent and 29.6 percent, respectively, and much lower male rates.

Some countries display moderate obesity rates for both sexes, though the gender variations remain evident. In Namibia, female obesity prevalence is 22.8 percent (confidence interval: 17.2 to 28.9 percent), while male prevalence is 10.2 percent (confidence interval:

5.4 to 16.3 percent). Mauritius and Cabo Verde follow similar patterns, with female rates of 25.3 percent and 22.9 percent, and male rates of 12.9 percent and 8.4 percent, respectively.

In countries such as Ghana, Cameroon, and Zimbabwe, female obesity prevalence ranges from 20 to 21 percent, while male rates are much lower, generally between 5 and 9 percent. For instance, in Ghana, female obesity is 20.3 percent (confidence interval: 17.0 to 23.8 percent), compared to 5.1 percent for men (confidence interval: 3.6 to 7.1 percent).

Some nations report relatively low obesity rates for both sexes, but the gender disparity persists. In Ethiopia, female obesity is just 4.5 percent (confidence interval: 3.2 to 6.1 percent), while male prevalence is even lower at 1.1 percent (confidence interval: 0.6 to 1.7 percent). Eritrea, Madagascar, and Rwanda also have low rates, with female obesity between 4 and 7.7 percent and male obesity below 5 percent.

A few countries display less pronounced gender differences. In Burundi and Madagascar, male and female obesity rates are relatively close, with male rates sometimes slightly higher or similar to female rates. For example, in Madagascar, female obesity is 4.1 percent (confidence interval: 3.0 to 5.5 percent), while male prevalence is 4.5 percent (confidence interval: 2.0 to 8.3 percent).

Moderate obesity prevalences are seen in countries such as Benin, Côte d'Ivoire, and Mozambique, where female rates range from 13.7 to 17.4 percent and male rates from 5.6 to 9.7 percent. These figures suggest emerging public health challenges related to obesity, particularly among women. This should be removed to discussion section as well as part of conclusion

# Table 2: Sex disaggregated obesity prevalence among adults in Africa, 2022

| **Year** | **Country** | **Sex** | **Estimate** | **Lower**  **Bound** | **Upper**  **Bound** |
| --- | --- | --- | --- | --- | --- |
| 2022 | South Africa | Female | 45.8 | 41.5 | 50.3 |
| 2022 | South Africa | Male | 13.9 | 11.1 | 17.1 |
| 2022 | Algeria | Female | 32.3 | 27.1 | 37.7 |
| 2022 | Algeria | Male | 15.5 | 11.6 | 20.0 |

| 2022 | Angola | Female | 16.3 | 9.9 | 24.2 |
| --- | --- | --- | --- | --- | --- |
| 2022 | Angola | Male | 6.3 | 2.9 | 11.3 |
| 2022 | Benin | Female | 15.0 | 12.0 | 18.2 |
| 2022 | Benin | Male | 7.1 | 4.6 | 10.4 |
| 2022 | Botswana | Female | 27.2 | 21.4 | 33.6 |
| 2022 | Botswana | Male | 8.4 | 5.1 | 12.8 |
| 2022 | Burkina Faso | Female | 9.3 | 7.7 | 11.0 |
| 2022 | Burkina Faso | Male | 4.0 | 2.8 | 5.5 |
| 2022 | Burundi | Female | 4.0 | 2.6 | 5.8 |
| 2022 | Burundi | Male | 6.1 | 2.1 | 12.9 |
| 2022 | Cabo Verde | Female | 22.9 | 19.2 | 26.7 |
| 2022 | Cabo Verde | Male | 8.4 | 6.2 | 11.0 |
| 2022 | Cameroon | Female | 20.4 | 17.0 | 23.9 |
| 2022 | Cameroon | Male | 9.3 | 6.1 | 13.0 |
| 2022 | Central African Republic | Female | 12.3 | 8.3 | 17.2 |
| 2022 | Central African Republic | Male | 6.1 | 3.7 | 9.3 |
| 2022 | Chad | Female | 6.0 | 4.0 | 8.7 |
| 2022 | Chad | Male | 7.4 | 3.5 | 13.1 |
| 2022 | Comoros | Female | 24.5 | 18.9 | 30.9 |
| 2022 | Comoros | Male | 8.0 | 4.7 | 12.6 |
| 2022 | Congo | Female | 12.8 | 8.8 | 17.3 |
| 2022 | Congo | Male | 4.2 | 2.1 | 7.3 |
| 2022 | Cote d'Ivoire | Female | 15.7 | 12.8 | 19.0 |
| 2022 | Cote d'Ivoire | Male | 7.7 | 4.1 | 12.6 |
| 2022 | Democratic Republic of the  Congo | Female | 9.0 | 6.2 | 12.1 |
| 2022 | Democratic Republic of the  Congo | Male | 4.2 | 2.3 | 6.9 |
| 2022 | Equatorial Guinea | Female | 29.6 | 21.9 | 37.8 |
| 2022 | Equatorial Guinea | Male | 7.8 | 2.7 | 16.2 |
| 2022 | Eritrea | Female | 6.6 | 3.9 | 10.3 |

| 2022 | Eritrea | Male | 2.8 | 1.3 | 5.2 |
| --- | --- | --- | --- | --- | --- |
| 2022 | Eswatini | Female | 43.9 | 37.2 | 50.7 |
| 2022 | Eswatini | Male | 15.0 | 10.4 | 20.4 |
| 2022 | Ethiopia | Female | 4.5 | 3.2 | 6.1 |
| 2022 | Ethiopia | Male | 1.1 | 0.6 | 1.7 |
| 2022 | Gabon | Female | 31.7 | 27.0 | 36.4 |
| 2022 | Gabon | Male | 11.0 | 5.8 | 17.8 |
| 2022 | Gambia | Female | 20.0 | 16.8 | 23.4 |
| 2022 | Gambia | Male | 9.5 | 5.5 | 14.9 |
| 2022 | Ghana | Female | 20.3 | 17.0 | 23.8 |
| 2022 | Ghana | Male | 5.1 | 3.6 | 7.1 |
| 2022 | Guinea | Female | 12.7 | 9.8 | 16.3 |
| 2022 | Guinea | Male | 5.9 | 3.0 | 10.1 |
| 2022 | Guinea-Bissau | Female | 14.8 | 10.0 | 20.7 |
| 2022 | Guinea-Bissau | Male | 7.6 | 2.9 | 15.4 |
| 2022 | Kenya | Female | 18.5 | 15.9 | 21.4 |
| 2022 | Kenya | Male | 6.0 | 4.6 | 7.7 |
| 2022 | Lesotho | Female | 32.7 | 26.9 | 38.8 |
| 2022 | Lesotho | Male | 8.4 | 5.5 | 12.0 |
| 2022 | Liberia | Female | 21.0 | 18.3 | 23.7 |
| 2022 | Liberia | Male | 12.8 | 10.3 | 15.6 |
| 2022 | Madagascar | Female | 4.1 | 3.0 | 5.5 |
| 2022 | Madagascar | Male | 4.5 | 2.0 | 8.3 |
| 2022 | Malawi | Female | 11.8 | 9.2 | 14.6 |
| 2022 | Malawi | Male | 3.1 | 1.9 | 4.7 |
| 2022 | Mali | Female | 12.9 | 10.1 | 16.2 |
| 2022 | Mali | Male | 9.9 | 5.9 | 15.1 |
| 2022 | Mauritania | Female | 35.0 | 30.2 | 40.1 |
| 2022 | Mauritania | Male | 8.9 | 4.1 | 15.9 |
| 2022 | Mauritius | Female | 25.3 | 20.0 | 30.7 |
| 2022 | Mauritius | Male | 12.9 | 9.0 | 17.5 |
| 2022 | Mozambique | Female | 13.7 | 10.0 | 18.0 |
| 2022 | Mozambique | Male | 6.2 | 3.7 | 9.6 |
| 2022 | Namibia | Female | 22.8 | 17.2 | 28.9 |
| 2022 | Namibia | Male | 10.2 | 5.4 | 16.3 |
| 2022 | Niger | Female | 7.6 | 6.0 | 9.5 |
| 2022 | Niger | Male | 4.4 | 3.1 | 6.0 |
| 2022 | Nigeria | Female | 16.5 | 13.5 | 19.8 |
| 2022 | Nigeria | Male | 8.2 | 5.9 | 10.8 |
| 2022 | Rwanda | Female | 7.7 | 6.4 | 9.3 |
| 2022 | Rwanda | Male | 1.8 | 1.2 | 2.6 |
| 2022 | Sao Tome and Principe | Female | 23.3 | 19.3 | 27.7 |
| 2022 | Sao Tome and Principe | Male | 9.7 | 6.9 | 12.8 |
| 2022 | Senegal | Female | 15.5 | 11.8 | 19.6 |
| 2022 | Senegal | Male | 3.9 | 2.3 | 6.0 |
| 2022 | Seychelles | Female | 39.6 | 31.8 | 47.4 |
| 2022 | Seychelles | Male | 20.2 | 13.7 | 27.5 |
| 2022 | Sierra Leone | Female | 11.4 | 8.9 | 14.4 |
| 2022 | Sierra Leone | Male | 2.7 | 1.8 | 3.8 |
| 2022 | South Sudan | Female | 11.8 | 6.8 | 18.4 |
| 2022 | South Sudan | Male | 5.1 | 2.4 | 9.2 |
| 2022 | Togo | Female | 17.4 | 14.7 | 20.3 |
| 2022 | Togo | Male | 5.6 | 4.2 | 7.3 |
| 2022 | Uganda | Female | 11.3 | 9.5 | 13.4 |
| 2022 | Uganda | Male | 4.1 | 3.1 | 5.3 |
| 2022 | United Republic of Tanzania | Female | 18.3 | 14.5 | 22.4 |
| 2022 | United Republic of Tanzania | Male | 6.5 | 3.9 | 9.9 |
| 2022 | Zambia | Female | 16.4 | 12.9 | 20.2 |
| 2022 | Zambia | Male | 5.1 | 3.2 | 7.4 |
| 2022 | Zimbabwe | Female | 20.9 | 16.4 | 26.1 |
| 2022 | Zimbabwe | Male | 5.5 | 3.4 | 8.0 |

354

# Disparity of sex disaggregated obesity prevalence among adults in Africa

Table 3 presents thedisparity in obesity prevalence between sexes in African countries for 2022, reported as the difference (D) between female and male obesity rates. Positive values indicate higher obesity prevalence among women, while negative values indicate higher prevalence among men. The results show that, with a few exceptions, obesity prevalence is consistently higher among women than men across the continent.

The largest disparities are observed in South Africa (D = 31.8), Eswatini (D = 28.8), Mauritania (D = 26.1), Lesotho (D = 24.2), Equatorial Guinea (D = 21.8), Seychelles (D = 19.4), Botswana (D = 18.8), Comoros (D = 16.5), Algeria (D = 16.8), Zimbabwe (D = 15.4), Ghana (D = 15.2), and Cabo Verde (D = 14.5). In these countries, the differences in obesity prevalences between women and men exceed 14 percentage points, with South Africa showing the most marked disparity. These figures highlight significant gender disparities, suggesting that women are much more likely to be obese than men, possibly due to a combination of biological, cultural, and socioeconomic factors that affect women disproportionately. This should be removed to discussion section as well as part of conclusion

Countries such as Namibia (D = 12.6), Mauritius (D = 12.4), Kenya (D = 12.5), Senegal (D = 11.6), United Republic of Tanzania (D = 11.8), Togo (D = 11.8), Zambia (D = 11.3), Cameroon (D = 11.1), and Gambia (D = 10.5) also exhibit substantial gender variations, with differences between 10 and 13 percentage points. This should be removed to discussion section as well as part of conclusion

A number of countries show moderate disparities, with differences ranging from 6 to 10 percentage points. These include Angola (D = 10.1), Liberia (D = 8.2), Nigeria (D = 8.3), Sierra Leone (D = 8.8), Malawi (D = 8.7), Congo (D = 8.6), Cote d'Ivoire (D = 8.0), Benin (D = 7.9), Mozambique (D = 7.5), Guinea-Bissau (D = 7.3), Uganda (D = 7.2), and Guinea (D = 6.8). While the gender disparities are less extreme in these countries, women still have noticeably higher prevalences of obesity than men.

Some countries, such as Central African Republic (D = 6.2), Rwanda (D = 6.0), Burkina Faso (D = 5.3), Democratic Republic of the Congo (D = 4.8), Eritrea (D = 3.8), Niger (D = 3.3), Mali

(D = 3.0), and Ethiopia (D = 3.5), show smaller disparities, with D values between 3 and 6 percentage points. In these countries, while the difference remains in favour of higher female obesity, the gap is relatively narrow. This should be removed to discussion section as well as part of conclusion

A few countries display negative or near-zero differences, indicating either a very small gender variations or, in rare cases, higher obesity prevalences among men. Burundi (D = -2.1), Chad (D = -1.4), and Madagascar (D = -0.4) are notable in this regard. In these countries, the obesity prevalence between men and women is either similar or slightly higher among men, which contrasts sharply with the general trend observed across the continent. These exceptions may reflect unique cultural, dietary, or economic circumstances. This should be removed to discussion section as well as part of conclusion

# Table 3: Disparity in sex disaggregated obesity prevalence among adults in Africa, 2022

| **Year** | **Country** | **Dimension** | **Measure** | **Estimate** |
| --- | --- | --- | --- | --- |
| 2022 | South Africa | Sex | Difference (D) | 31.8 |
| 2022 | Algeria | Sex | Difference (D) | 16.8 |
| 2022 | Angola | Sex | Difference (D) | 10.1 |
| 2022 | Benin | Sex | Difference (D) | 7.9 |
| 2022 | Botswana | Sex | Difference (D) | 18.8 |
| 2022 | Burkina Faso | Sex | Difference (D) | 5.3 |
| 2022 | Burundi | Sex | Difference (D) | -2.1 |
| 2022 | Cabo Verde | Sex | Difference (D) | 14.5 |
| 2022 | Cameroon | Sex | Difference (D) | 11.1 |
| 2022 | Central African Republic | Sex | Difference (D) | 6.2 |
| 2022 | Chad | Sex | Difference (D) | -1.4 |
| 2022 | Comoros | Sex | Difference (D) | 16.5 |
| 2022 | Congo | Sex | Difference (D) | 8.6 |
| 2022 | Cote d'Ivoire | Sex | Difference (D) | 8.0 |
| 2022 | Democratic Republic of the Congo | Sex | Difference (D) | 4.8 |
| 2022 | Equatorial Guinea | Sex | Difference (D) | 21.8 |
| 2022 | Eritrea | Sex | Difference (D) | 3.8 |
| 2022 | Eswatini | Sex | Difference (D) | 28.8 |
| 2022 | Ethiopia | Sex | Difference (D) | 3.5 |
| 2022 | Gabon | Sex | Difference (D) | 20.7 |
| 2022 | Gambia | Sex | Difference (D) | 10.5 |
| 2022 | Ghana | Sex | Difference (D) | 15.2 |
| 2022 | Guinea | Sex | Difference (D) | 6.8 |
| 2022 | Guinea-Bissau | Sex | Difference (D) | 7.3 |
| 2022 | Kenya | Sex | Difference (D) | 12.5 |
| 2022 | Lesotho | Sex | Difference (D) | 24.2 |
| 2022 | Liberia | Sex | Difference (D) | 8.2 |
| 2022 | Madagascar | Sex | Difference (D) | -0.4 |
| 2022 | Malawi | Sex | Difference (D) | 8.7 |
| 2022 | Mali | Sex | Difference (D) | 3.0 |
| 2022 | Mauritania | Sex | Difference (D) | 26.1 |
| 2022 | Mauritius | Sex | Difference (D) | 12.4 |
| 2022 | Mozambique | Sex | Difference (D) | 7.5 |
| 2022 | Namibia | Sex | Difference (D) | 12.6 |
| 2022 | Niger | Sex | Difference (D) | 3.3 |
| 2022 | Nigeria | Sex | Difference (D) | 8.3 |
| 2022 | Rwanda | Sex | Difference (D) | 6.0 |
| 2022 | Sao Tome and Principe | Sex | Difference (D) | 13.6 |
| 2022 | Senegal | Sex | Difference (D) | 11.6 |
| 2022 | Seychelles | Sex | Difference (D) | 19.4 |
| 2022 | Sierra Leone | Sex | Difference (D) | 8.8 |
| 2022 | South Sudan | Sex | Difference (D) | 6.6 |
| 2022 | Togo | Sex | Difference (D) | 11.8 |
| 2022 | Uganda | Sex | Difference (D) | 7.2 |
| 2022 | United Republic of Tanzania | Sex | Difference (D) | 11.8 |
| 2022 | Zambia | Sex | Difference (D) | 11.3 |
| 2022 | Zimbabwe | Sex | Difference (D) | 15.4 |

# Discussion

This study presents a comprehensive assessment of adult obesity prevalence and sex based disparities across African countries, revealing substantial heterogeneity in both overall rates and gender differences. The findings highlight that while obesity remains a pressing and growing public health concern in Africa, its distribution is far from uniform, with some countries facing far greater burdens than others. Most notably, the results demonstrate that women are disproportionately affected by obesity in nearly every country, with the gender variations being particularly wide in Southern and some Western African nations. These patterns underscore the need for nuanced, context-specific interventions and policies to address the continent’s evolving obesity epidemic.

The analysis shows that South Africa has the highest prevalence of adult obesity in Africa, with nearly one-third of adults classified as obese. Eswatini and Seychelles also report rates close to 30 percent, while several other countries, including Algeria, Mauritania, Lesotho, Gabon, and Botswana, have prevalence rates exceeding 18 percent. In contrast, countries such as Ethiopia, Eritrea, Rwanda, Madagascar, and Burundi report much lower rates, often below 5 percent. This clear north-south and east-west divide in obesity prevalence aligns with previous studies that have documented substantial regional variation in noncommunicable disease risk factors across Africa [9,19].

A central finding of this study is the marked sex disparity in obesity prevalence. Across nearly all countries, women have significantly higher rates of obesity than men. In South Africa, the difference exceeds 30 percentage points, and in countries such as Eswatini, Mauritania, Lesotho, Equatorial Guinea, and Seychelles, the gap is similarly wide. Only a few countries, such as Burundi, Chad, and Madagascar, show negligible or slightly negative differences, indicating either parity or higher obesity prevalence among men. These findings are consistent with global trends but are even more pronounced in the African context [20,21].

The observed patterns in obesity prevalence and gender disparities can be attributed to a complex interplay of sociocultural, economic, and biological factors. Rapid urbanization shifts in dietary patterns toward energy-dense, nutrient-poor foods, and reduced physical activity have all contributed to rising obesity rates in many African countries [22]. Countries with the highest prevalences, such as South Africa, Eswatini, and Seychelles, have experienced remarkable lifestyle changes over recent decades, including increased consumption of processed foods and sedentary behaviours [23,24].

The consistently higher rates of obesity among women may be partially explained by sociocultural norms that associate higher body weight with prosperity, health, or attractiveness in certain African societies [25,26]. These cultural perceptions may contribute to greater acceptance or even encouragement of overweight and obesity among women, while men may face different societal pressures regarding body image. Additionally, women often have less access to physical activity opportunities and may experience greater barriers to healthy eating, including lower socioeconomic status and limited autonomy over food choices [10].

Biological factors, such as hormonal differences and the effects of pregnancy and childbirth, may also play a role in the higher prevalence of obesity among women [27]. Furthermore, women in many African countries are more likely to experience weight gain during and after pregnancy and may have fewer resources or social support for postpartum weight management [28].

The few countries where the gender gap is negligible or reversed such as Burundi, Chad, and Madagascar may reflect unique local circumstances, such as prevailing food insecurity, high levels of physical labour among both sexes, and different cultural attitudes toward body weight [29]. These exceptions highlight the importance of context-specific research and interventions.

# Implications for policy and practice

The findings of this study have important implications for public health policy and practice in Africa. First, the high and rising prevalence of obesity in several countries underscores the urgent need for comprehensive, multisectoral interventions that address the root causes of obesity. Policies should focus on promoting healthy diets, increasing opportunities for physical activity, and reducing the marketing and availability of unhealthy foods, particularly in urban areas [30,31]. Given the pronounced gender disparities, interventions must be tailored to address the specific needs and challenges faced by women. This includes empowering women with nutrition education, improving access to affordable healthy foods, and creating supportive environments for physical activity. Programs that engage communities and challenge cultural norms around body weight may be particularly effective in shifting attitudes and behaviours [32]. At the same time, countries with lower obesity prevalence should not become complacent. As economic development and urbanization continue, these countries may also experience rising rates of obesity and related non-communicable diseases. Early preventive action could help mitigate future health burdens.

# Strengths and limitations of this study

This study has several strengths, including the use of recent, nationally representative, and sex-disaggregated data from 47 African countries, which enhances the generalizability of the findings across the continent. The application of the WHO Health Equity Assessment Toolkit provided standardized data collection and facilitated comparability between countries. However, the cross-sectional design allows only for the assessment of prevalence and sex disparities and does not enable causal inferences regarding the determinants of obesity. The analysis was limited to countries and years with available, high-quality data, which may result in the underrepresentation of certain regions or recent trends. Additionally, the lack of adjustment for potential confounders such as age distribution, socioeconomic status, and urban-rural differences may affect the interpretation of between-country differences.

# Future directions

Future research should aim to collect and analyse longitudinal data to better understand trends and determinants of obesity in Africa. Studies that incorporate more granular data on socioeconomic, behavioural, and environmental factors will help elucidate the pathways linking these variables to obesity risk. In addition, qualitative research exploring cultural attitudes toward body weight and health could inform the design of more effective, culturally sensitive interventions. There is also a need for intervention studies that evaluate the effectiveness of different policy and programmatic approaches to obesity prevention and control in diverse African contexts.

# Conclusions

This study provides a detailed picture of the landscape of adult obesity in Africa, revealing substantial variations in prevalences both across countries and between sexes. The findings highlight that women are disproportionately affected by obesity in nearly all African countries, with the gender variations being particularly wide in Southern and Western Africa. These patterns reflect the combined influence of sociocultural, economic, and biological factors and underscore the need for targeted, context-specific interventions. Addressing the obesity epidemic in Africa will require coordinated action across sectors, with particular attention to the unique challenges faced by women. Early and sustained efforts to promote healthy lifestyles and environments are essential to curbing the rising tide of obesity and its associated health consequences on the continent.

# Declarations Author Contributions

AO contributed to the conceptualisation of the article, drafted the original manuscript and led the review and editing of subsequent drafts. AO approved the final version of the manuscript. AO is the guarantor and accepts full responsibility for the overall content of the work.

# Funding

The authors have not declared a specific grant for this research from any funding agency in the public, commercial or not- for- profit sectors.

**Competing interests** None declared.

# Availability of data and materials

The data used in this study was obtained from the World Health Organization Global Health Observatory (WHO GHO) and the WHO Health Equity Assessment Toolkit (HEAT). These data are publicly available and can be accessed without restriction from the WHO Global Health Observatory website (https://www.who.int/data/gho) and the WHO HEAT platform (https://www.who.int/data/inequality-monitor/assessment_toolkit). The author confirm that all interested researchers can access these data in the same manner as the authors, and no special access privileges were required.

# References

1. World Health Organization. Obesity and overweight. Geneva: WHO; 2024. Available from: https://www.who.int/news-room/fact-sheets/detail/obesity-and-overweight 2. GBD 2015 Obesity Collaborators. Health Effects of Overweight and Obesity in 195 Countries over 25 Years. N Engl J Med. 2017;377(1):13-27.

3. Hruby A, Hu FB. The Epidemiology of Obesity: A Big Picture. Pharmacoeconomics. 2015;33(7):673-89.
4. Popkin BM, Corvalan C, Grummer-Strawn LM. Dynamics of the double burden of malnutrition and the changing nutrition reality. Lancet. 2020;395(10217):65-74.
5. Bowo-Ngandji A, Kenmoe S, Ebogo-Belobo JT, Kenfack-Momo R, Takuissu GR, KengneNde C, Mbaga DS, Tchatchouang S, Kenfack-Zanguim J, Lontuo Fogang R, Zeuko’o Menkem E. Prevalence of the metabolic syndrome in African populations: A systematic review and meta-analysis. PLoS One. 2023 Jul 27;18(7):e0289155.
6. World Health Organization. Obesity and overweight: Key facts. Geneva: WHO; 2024. Available	from:	https://www.who.int/news-room/fact-sheets/detail/obesity-andoverweight
7. McLachlan S, NCD Risk Factor Collaboration. Trends in adult body mass index in 200 countries since 1975: pooled analysis of 1,698 population-based measurement studies with

19.2 million participants. The Lancet. 2016 Apr 2;387(10026).

8. Case A, Menendez A. Sex differences in obesity rates in poor countries: Evidence from South Africa. Econ Hum Biol. 2009;7(3):271-82.
9. World Health Organization. Noncommunicable Diseases Country Profiles 2022. Geneva: WHO; 2022.
10. Ziraba AK, Fotso JC, Ochako R. Overweight and obesity in urban Africa: A problem of the rich or the poor? BMC Public Health. 2009; 9:465.
11. Dinsa GD, Goryakin Y, Fumagalli E, Suhrcke M. Obesity and socioeconomic status in developing countries: a systematic review. Obes Rev. 2012;13(11):1067-79.
12. Neupane S, Prakash KC, Doku DT. Overweight and obesity among adults in sub-Saharan Africa: a systematic review and meta-analysis. BMJ-Open. 2016;6(9): e010679.
13. Amugsi DA, Dimbuene ZT, Mberu B, Muthuri S, Ezeh AC. Prevalence and time trends in overweight and obesity among urban women: an analysis of demographic and health surveys data from 24 African countries, 1991–2014. BMJ Open. 2017;7(10): e017344.
14. Agyemang C, Boatemaa S, Frempong GA, de-Graft Aikins A. Obesity in Sub-Saharan

Africa. In: Ahima RS, editor. Metabolic Syndrome: A Comprehensive Textbook. Cham: Springer; 2016. p. 41-53.

15. Keino S, Plasqui G, Ettyang G, van den Borne B. Determinants of stunting and overweight among young children and adolescents in sub-Saharan Africa. Food Nutr Bull. 2014;35(2):167-78.
16. Holdsworth M, Gartner A, Landais E, Maire B, Delpeuch F. Perceptions of healthy and desirable body size in urban Senegalese women. Int J Obes Relat Metab Disord.

2004;28(12):1561-8.

17. Kruger HS, Puoane T, Senekal M, van der Merwe MT. Obesity in South Africa: challenges for government and health professionals. Public Health Nutr. 2005;8(5):491-500.
18. Sartorius B, Veerman LJ, Manyema M, Chola L, Hofman K. Determinants of obesity and associated population attributability, South Africa: Empirical evidence from a national panel survey, 2008-2012. PLoS One. 2015;10(6): e0130218.

[19]. World Health Organization. Health Equity Assessment Toolkit (HEAT): software for exploring and comparing health inequalities in countries. Geneva: WHO; 2021.

20. Ng M, Fleming T, Robinson M, Thomson B, Graetz N, Margono C, Mullany EC, Biryukov S, Abbafati C, Abera SF, Abraham JP. Global, regional, and national prevalence of overweight and obesity in children and adults during 1980–2013: a systematic analysis for the Global Burden of Disease Study 2013. The lancet. 2014 Aug 30;384(9945):766-81.
21. Musaiger AO. Overweight and obesity in eastern mediterranean region: prevalence and possible causes. Journal of obesity. 2011;2011(1):407237.
22. Popkin BM, Du S. Dynamics of the nutrition transition toward the animal foods sector in China and its implications: a worried perspective. The Journal of nutrition. 2003 Nov 1;133(11):3898S-906S.
23. Steyn NP, Bradshaw D, Norman R, Joubert JD, Schneider M, Steyn K. Dietary changes and the health. The double burden of malnutrition: Case Studies from six developing countries. 2006; 84:259.
24. Prentice AM. The emerging epidemic of obesity in developing countries. Int J Epidemiol. 2006;35(1):93–99.
25. Balogun-Mwangi O. Embracing the Hottentot Venus: A mixed-methods examination of body image, body image ideals and objectified body consciousness among African women. Northeastern University; 2016.
26. Puoane T, Fourie JM, Shapiro M, Rosling L, Tshaka NC, Oelefse A. 'Big is beautiful'-an exploration with urban black community health workers in a South African township. South African Journal of Clinical Nutrition. 2005 Apr 1;18(1):6-15.
27. Lovejoy JC, Sainsbury A, Stock Conference 2008 Working Group. Sex differences in obesity and the regulation of energy homeostasis. Obesity reviews. 2009 Mar;10(2):154-67. 28. Keetile M, Navaneetham K, Letamo G, Rakgoasi SD. Socioeconomic inequalities in noncommunicable disease risk factors in Botswana: a cross-sectional study. BMC Public Health. 2019 Dec; 19:1-9.
29. NCD Risk Factor Collaboration. Trends in adult body-mass index in 200 countries from 1975 to 2014: a pooled analysis of 1698 population-based measurement studies with 19· 2 million participants. Lancet (London, England). 2016 Apr 2;387(10026):1377.
30. World Health Organization. Global Action Plan for the Prevention and Control of Noncommunicable Diseases 2013–2020. Geneva: WHO; 2013.
31. Swinburn BA, Sacks G, Hall KD, McPherson K, Finegood DT, Moodie ML, Gortmaker SL. The global obesity pandemic: shaped by global drivers and local environments. The lancet. 2011 Aug 27;378(9793):804-14.
32. Alaba O, Chola L. Socioeconomic inequalities in adult obesity prevalence in South Africa: a decomposition analysis. International journal of environmental research and public health. 2014 Mar;11(3):3387-406.
