# Supplementary material for: Sex disparities in adult obesity prevalence across 47 African countries: A cross-sectional descriptive study
Source: PLoS One. 2025 Dec 22;20(12):e0339595. doi: 10.1371/journal.pone.0339595 (PMC12721531; doi:10.1371/journal.pone.0339595)
Supplement: Attachment — Submitted filename: Response to Reviewers.docx [file pone.0339595.s003.docx]

Journal requirements:

When submitting your revision, we need you to address these additional requirements.

1. Please ensure that your manuscript meets PLOS ONE's style requirements, including those for file naming. The PLOS ONE style templates can be found at

<https://journals.plos.org/plosone/s/file?id=wjVg/PLOSOne_formatting_sample_main_body.pdf> and

<https://journals.plos.org/plosone/s/file?id=ba62/PLOSOne_formatting_sample_title_authors_affiliations.pdf>

Response: I have revised the manuscript to comply with PLOS ONE’s formatting requirements. The file name follows the prescribed convention, and both the main text and title/author/affiliation pages use the journal’s style templates as provided in the links.

2. We suggest you thoroughly copyedit your manuscript for language usage, spelling, and grammar.

Response: I have carefully copyedited the manuscript to improve language usage, spelling, and grammar throughout the text.

Additional Editor Comments:

Please ensure that you address all the comments of both reviewers and provide a point-by-point explanation of the changes you made to the revised manuscript. Additionally, please ensure that all changes are marked in track change in the revised manuscript. Thank you.

Response: All reviewer comments have been addressed in a point-by-point manner below. All changes are marked using track changes in the revised manuscript.

Reviewer #1: Specific comments:
1. The study TITLE need not reflect the WHO tool utilized for the study. It suffices to be in the methodology

Response: Revised the title to remove the WHO tool reference. New title: “Sex disparities in adult obesity prevalence across 47 African countries: a cross-sectional descriptive study.” I have now retained full details of HEAT in Methods (Data source).

2. Tables are to be properly labelled and should not spill into pages

Response: Reformatted Tables 1–3 to fit on single pages, simplified column structure, standardized captions, and added footnotes. I also ensured consistent font, spacing, and alignment. Each table now includes a clear title and footnotes explaining abbreviations/measures.
3. Technical epidemiological terms such as significant (may connote statistical test), gaps (may connote unmet needs) have been inappropriately used in the text. Alternatives have been included in the review.

Response: Replaced “significant” with “marked,” “substantial,” or “pronounced” where no statistical test was performed; replaced “gaps” with “disparities” or “differences” as appropriate. These edits have been made throughout Abstract, Results, and Discussion. I also deleted any phrases implying hypothesis testing.

4. Elements of discussion should not come up in the results section but taken to the appropriate sections of discussion and conclusion. These have been indicated (green highlights) in situ of this review.

Response: Removed interpretive statements from Results and moved them to Discussion and/or Conclusion. Specifically, content identified in the review (highlighted in green) and lines corresponding to current manuscript narrative have been relocated. The Results now strictly present descriptive findings.

5. A separate subsection to make concrete actionable stakeholder - targeted recommendations is needed

Response: Added a new subsection “Policy and practice recommendations” at the end of Discussion. It includes stakeholder-specific, actionable steps for ministries of health, finance, urban planning, education, and civil society, with examples (e.g., SSB taxes, front-of-pack labelling, marketing restrictions, active transport).

Reviewer #2: This manuscript addresses an important and timely public health issue. The topic is highly relevant to the journal's readership, given the rising burden of obesity across Africa and the urgent need for equity-focused interventions.

Strengths

• The study covers a wide geographic scope (47 countries) and utilizes a standardized, validated, and publicly accessible dataset (WHO HEAT), enhancing transparency and reproducibility.

Response: Thank you.

• The presentation of sex-disaggregated data across African countries fills an important gap in the literature and contributes meaningfully to equity-focused obesity research in the region.

Response: Thank you.

Areas for Improvement

Introduction

1. The Introduction would benefit from substantial reorganization to improve its logical flow and coherence. At present, it mixes global, LMIC, and Africa-specific information without a clear structure and alternates between describing the causes and consequences of obesity, which makes the narrative difficult to follow. I recommend restructuring this section to follow a clear, progressive “funnel” logic, starting with the global burden and health consequences, then narrowing to the African context, followed by sex disparities, data gaps, and finally, the study’s aims and rationale. Adopting this structure will greatly improve clarity, readability, and scientific framing.

Response: Rewrote the Introduction following a funnel structure: (a) global burden and health consequences, (b) narrowing to LMICs, (c) African context, (d) sex disparities, (e) data gaps/limitations in existing literature, (f) study aims/rationale. We removed redundancies and improved coherence.

Methods

2. In the Data Source section [lines 182–192], please include the link to the WHO HEAT platform to improve transparency and reproducibility.

Response: Added direct links to both WHO HEAT and WHO GHO in the Data source subsection: https://www.who.int/data/inequality-monitor/assessment_toolkit and https://www.who.int/data/gho.

3. In the Inclusion and Exclusion Criteria section [lines 194-198], specify which countries were excluded and clarify whether the 47 included countries correspond to those within the WHO AFRO region. This will allow for a more informed discussion of coverage and missing data.

Response: Specified which countries were excluded due to missing sex-disaggregated data and clarified that the 47 countries correspond to WHO African Region coverage where data were available as of 2022.

4. The manuscript mentions that results are presented “in both tabular and graphical formats,” but no figures are shown. Including selected visualizations (e.g., bar charts or scatterplots) in the Results section would make the findings more engaging and easier to interpret.

Response: Reformatted Tables 1–3 to fit on single pages, simplified column structure, standardized captions, and added footnotes. I also ensured consistent font, spacing, and alignment. Each table now includes a clear title and footnotes explaining abbreviations/measures.

5. The analysis is entirely descriptive and cross-sectional. While appropriate for the study’s objectives, adding simple contextual analyses (e.g., correlation of obesity prevalence with socioeconomic or urbanization indicators) would strengthen the explanatory depth and policy relevance.

Response: I appreciate this suggestion. In addition to the descriptive, cross-sectional analyses, I have now included simple contextual analyses to enhance interpretability. I have reference these findings in the Discussion to link observed patterns to structural and commercial determinants.

Results
6. The Results section provides valuable data from 47 countries; however, it would benefit from clearer presentation and sharper analytical focus. Some interpretive statements (e.g., lines 281–293) belong in the Discussion rather than in the Results.

Response: Cleaned Results to be descriptive only; moved interpretive content to Discussion per your guidance.

7. Table presentation: Simplify tables to improve readability. Remove repetitive elements (such as the year (2022), if constant across all data) and merge duplicate country names. In Table 3, redundant columns (e.g., “dimension” and “sex”) could be removed, and the difference (D) defined in a table footnote.

Response: Reformatted Tables 1–3 to fit on single pages, simplified column structure, standardized captions, and added footnotes. I also ensured consistent font, spacing, and alignment. Each table now includes a clear title and footnotes explaining abbreviations/measures.

8. Data ordering: Arrange countries in descending order of prevalence to easily highlight the top and bottom 10 countries, making trends easier to identify.

Response: Ordered Tables 1–3 accordingly. Added a table footnote and text in Results identifying top and bottom 10 countries.

9. Analytical depth: Include simple summary measures such as the median female–male difference or the number of countries with higher female prevalence.

Response: I have now included a summary measure median female–male difference in the manuscript.

10. Visual presentation: Add visual elements such as bar charts or heatmaps of obesity prevalence by sex.

Response: Thank you for the suggestion to enhance visual presentation. To preserve consistency with our journal’s format and the study’s emphasis on comparability, I have retained the primary results in tables.

11. Socioeconomic analysis: A brief comparison of obesity prevalence by income group or GDP level would enhance interpretation and set up a stronger discussion of socioeconomic and structural determinants.

Response: Thank you for this helpful suggestion. I have added a brief socioeconomic comparison in the Results using World Bank income groups for the 47 countries. Specifically, I report summary measures of the female–male difference (D) and note that upper-middle-income countries exhibit higher median adult obesity prevalence and larger median female–male gaps than lower-income peers. I also summarize counts (e.g., number of countries with D ≥ 20 pp) by income group. These additions set up and are referenced in the revised Discussion, where I interpret the observed gradient in light of structural and commercial determinants (e.g., ultra-processed food penetration, marketing intensity, and urban environments).

Discussion

12. The author(s) should more clearly separate data interpretation from result description e.g. lines 410-421 [which belongs to the results section].

Response: Revised Discussion to focus on interpretation, mechanisms, policy implications, and comparisons to literature. Any descriptive result statements that belong in Results were moved.
13. The discussion of determinants is narrow. Expanding on structural and environmental drivers, such as the food environment, marketing of unhealthy foods, and weak fiscal or regulatory responses would position the findings within the broader commercial determinants of health framework. Reference to existing African policy examples (e.g., South Africa’s sugar-sweetened beverage tax or WHO’s recommendations on food marketing) would strengthen the discussion.

Response: Added a subsection on commercial determinants of health, food environments, marketing of ultra-processed foods, and weak regulatory responses; cited recent WHO recommendations and African examples, including South Africa’s SSB tax (2018) with emerging evidence, and other policy levers (marketing restrictions, front-of-pack labelling, fiscal measures).

14. The equity framing should go beyond sex differences to include socioeconomic and geographic inequalities, reflecting the full potential of the WHO HEAT tool.

Response: Expanded the equity framing to note the importance of socioeconomic status, urban–rural residence, and subnational variation. Clarified that while this study focuses on sex due to data availability within HEAT, the tool supports other equity dimensions for future analyses. Added this to both Discussion and Future directions.

15. Comparative context would enhance interpretation, drawing on examples from other LMICs or regions to highlight similarities or contrasts in obesity trends.

Response: Added a paragraph comparing observed patterns to Latin America, Middle East/North Africa, and Pacific Islands literature, noting similarities/differences in sex disparities and policy approaches.

16. Regarding biological explanations (lines 445-449), please clarify whether hormonal or reproductive factors significantly account for observed sex differences, as these factors are universal (present in countries with low obesity prevalence as well) and may not fully explain inter-country variation.

Response: Clarified that while biological factors may contribute to sex differences, they are unlikely to explain cross-country heterogeneity; social, cultural, and environmental factors likely play a larger role in between-country variation. Revised language accordingly.

17. Policy recommendations should be more specific and actionable.

Response: Added a detailed “Policy and practice recommendations” subsection with prioritized, feasible, and stakeholder-specific actions; included examples, timelines, and responsible actors in narrative form.

18. Finally, while limitations are noted [lines 481-485], they have not acknowledged that HEAT relies on secondary data sources with variable quality across countries.

Response: Added this explicit limitation, discussed implications for cross-country comparability, and referenced variability in survey rounds, sampling, and measurement approaches.

19. Several references (e.g., 16, 17, 24, 26) are dated. The author(s) are encouraged to include more recent and relevant literature to strengthen the evidence base and ensure the discussion reflects current research.

Response: Added recent WHO policy documents (2022–2024), new studies on obesity trends and commercial determinants in Africa, and updated evidence on SSB taxes and food marketing restrictions. Retained foundational references where still appropriate.

Recommendation

This manuscript makes a valuable contribution to the understanding of obesity in Africa and highlights important sex disparities. However, its current form limits its scientific and policy impact. I recommend major revision before consideration for publication. With the above improvements, along with careful proofreading for language and consistency, the manuscript has strong potential to make a meaningful and policy-relevant contribution to obesity research and public health practice in Africa.

Response: Thank you for your constructive feedback and recommendation for major revision. I have addressed all points as detailed above, and carefully proofread the manuscript for language and consistency. I appreciate the opportunity to improve the manuscript and believe these revisions substantially enhance its scientific and policy relevance.
